# Agriculture, bioenergy, and water implications of constrained cereal trade and climate change impacts

**DOI:** 10.1371/journal.pone.0291577

**Published:** 2023-09-15

**Authors:** Ying Zhang, Stephanie Waldhoff, Marshall Wise, Jae Edmonds, Pralit Patel

**Affiliations:** Joint Global Change Research Institute, Pacific Northwest National Laboratory, College Park, Maryland, United States of America; University of Agriculture Faisalabad, PAKISTAN

## Abstract

International trade increases connections and dependencies between countries, weaving a network of global supply chains. Agricultural commodity trade has implications for crop producers, consumers, crop prices, water and land uses, and other human systems. Interconnections among these systems are not always easy to observe when external impacts penetrate across multiple sectors. To better understand the interactions of non-linear and globally coupled agricultural-bioenergy-water systems under the broader economy, we introduce systematic perturbations in two dimensions, one human (restrictions on agricultural trade) and the other physical (climate impacts on crop yields). We explore these independently and in combination to distinguish the consequences of individual perturbation and interactive effects in long-term projections. We show that most regions experience larger changes in cereal consumption due to cereal import dependency constraints than due to the impacts of climate change on agricultural yields. In the scenario where all regions ensure an import dependency ratio of zero, the global trade of cereals decreases ~50% in 2050 compared to the baseline, with smaller decreases in cereal production and consumption (4%). The changes in trade also impact water and bioenergy: global irrigation water consumption increases 3% and corn ethanol production decreases 7% in 2050. Climate change results in rising domestic prices and declining consumption of cereal crops in general, while the import dependency constraint exacerbates the situation in regions which import more cereals in the baseline. The individual and interactive effects of trade perturbations and climate change vary greatly across regions, which are also affected by the regional ability to increase agricultural production through intensification or extensification.

## 1 Introduction

International trade and global supply chains create strong connections across regions. Trade in agricultural commodities impacts crop producers, consumers, crop prices, water and land uses, as well as other human systems. The potentially far-reaching implications of the combined effects of climate change impacts on crop yields and human decision-making have many interactions and feedbacks that can be difficult to disentangle, because all that is observed is the final consequence of the coupled systems.

The COVID-19 pandemic and recent extreme weather events [1] have highlighted the potential for disruptions to global supply chains when countries close borders or prioritize domestic demand [2–6]. In 2021, a massive container ship blocked the Suez Canal for nearly a week, causing waves of disruptions and delays on global supply chains, ranging from shortages of oil and natural gas supply in Europe to coffee and toilet paper shortages in the US [7, 8]. This effect of blockage at Suez Canal is transmitted to regions globally via trade, with continuing interactions with different sectors. While trade can benefit both importers and exporters, importing countries are vulnerable to disruptions in world markets [9]. Supply disruptions and domestic market reconfigurations can alter trade patterns, as was seen with the pandemic [10, 11] and the 2007–08 international food crisis [12, 13].

Climate change impacts on crop yields are heterogeneous across countries and regions, with the effect of changes in one country transmitted to others via trade. Although studies have generally shown net negative impacts on yields at a global level, some regions will experience improved yields while others have losses, changing the comparative advantage of current agricultural producers and trade dynamics [14–19].

International trade could play a role in mitigating the negative regional consequences of climate change impacts on crop yields. Previous studies have analyzed agricultural welfare loss under climate change and trade regimes at a global scale [20, 21]. They found integrated world markets largely buffer the adverse food security impacts due to climate change. However, Costinot, Donaldson [15] concluded that international trade plays a minor role in reducing climate impacts on global GDP, though this conclusion has been criticized due to the high elasticities of food supply and demand assumed in the model [22].

To better understand the interactions of non-linear and globally coupled agricultural-bioenergy-water systems under the broader economy, and in response to agricultural trade and climate change impacts, we introduce systematic perturbations to a global integrated human-Earth system model in two dimensions. First we explore the role of international trade patterns via cereal import dependency constraints, as a change in the human system dimension. Second, we examine changes to the Earth system via climate change impacts on crop yields. Perturbations are introduced both separately and in combination, allowing us to distinguish the consequences of individual perturbations and interactive effects.

We use the Global Change Analysis Model [GCAM v5.3; 23], a global, fully integrated model of the socioeconomic, energy, land, and water systems, to disentangle the individual roles of national and regional engagement in global markets and climate change and the non-linear interactions between these two systems over the period of 2015–2050. We track a set of multi-sector outcomes—crop production and consumption, exports, imports, crop prices, caloric consumption, agricultural revenue, net trade revenue, the food expenditure share of income, irrigation water consumption, and crop-based ethanol production.

We find that decreased participation in global markets due to all regions implementing a zero cereal import dependency strategy leads to decreases in total cereal production and consumption at the global scale. Although some regions show increases in production or consumption, no region shows increases in both. The impact of reduced international trade on production and consumption of cereals is stronger than climate change in most regions. The influence of climate change on crop yields also has strong impacts across regions, with rising domestic prices and declining consumption of cereal crops in general. Enforcement of a zero import-dependency for cereal crops exacerbates the effects of climate change in regions with higher baseline import dependence. Other outcomes, such as trade and crop producers’ revenue, irrigation water consumption, and crop-based ethanol production, are more sensitive to the resulting changes in international trade due to zero import-dependence than to climate change, with the largest effects displayed in regions with large share of base-year cereal imports. We intentionally set the global zero cereal import dependency constraint as a thought experiment to explore the impact of global trade restrictions on multi-sector outcomes. We are aware that this extreme experiment may not occur in the real world; however, our objective is not to perform a specific policy analysis but rather to analyze and understand the linkages embedded in the coupled systems using this experiment.

## 2 Materials and methods

GCAM version 5.3, available at https://github.com/JGCRI/gcam-core/releases, is modified and employed in this study. GCAM is an open-source global-scale integrated model that captures economic decisions and dynamic interactions between multiple systems. It is a market equilibrium model, in which the prices for all markets are solved simultaneously, such that supply equals demand. The model is well validated and documented with carefully designed model structure, open-source data inputs and model codes that support reproducibility. The model calibration periods span from 1975 to 2015. The final calibration year, 2015, is also called the base year, from which projections at 5-year time steps are made through 2100 to model long-term trends of different system outcomes. The model operates at 32 geopolitical regions for energy and economic systems overlayed with 235 water basins, resulting in 384 regions for water and land. Land use for competing purposes (e.g., crop land versus forest and land allocation among different crops) with price-induced intensification (e.g., irrigation) are included in the model’s market equilibrium to balance against agricultural demand (e.g., crop demand) with endogenously solved prices and quantities. International trade for agricultural commodities (including crops) among the 32 geopolitical regions is modeled with regionally differentiated markets (i.e., different regions can have different exporting prices for the same commodity). and Armington-style preferences between domestic and imported commodities [24, 25]. Regional exports and imports are linked through one global trading pool, where regional trading flows into and out of the pool (i.e., export to and import from the pool) are traced for each agricultural commodity. Thus, the global importing price reflects the producer prices from each exporting region weighted by the exporting quantities and all regions import from the same global pool with the same global price. We do not model the bi-lateral import and export explicitly in this study.

GCAM v5.3 models 11 aggregate crop commodities, including four cereal commodities (i.e., corn, rice, wheat, and other grains) and seven other crop commodities, which cover all Food and Agriculture Organization (FAO) reported crops (See S1 Table in S1 File for a list of GCAM agricultural commodities). The food demand model is derived based on neoclassical economics, which emphasizes the importance of consumers’ utility when economic decisions are made (e.g., purchasing food). The responses of food demand in the model to changes of consumer income and commodity price (i.e., income and price elasticities) evolve as income varies. In this model, demand is modeled for staple and non-staple foods. Staple food demand is supplied by caloric consumption of crops including cereals as well as roots and tubers, while consumption of all other agricultural commodities for food contributes to non-staple food demand. The food demand model is calibrated and cross-validated to empirical observations [26].

To introduce a perturbation to the human international trade system, we enforce a net zero cereal import requirement in GCAM, measured by the cereal import dependency ratio [CIDR; 27], which is the ratio of domestic net import over the sum of domestic production and the net import of cereals (Eq 1). The ratio can be deduced as one minus the ratio of domestic production over domestic consumption of cereals in total (Eq 2). Under global cereal import dependency constraints, we enforce the total of cereal production (in metric tons) in a region to be equal to or greater than the total of cereal consumption in the region, regardless of whether trade happens or not (i.e., CIDR≤0). Note that in our model, crop consumption includes food, non-food, bioenergy, feed, and others (e.g., wastes). As we are not looking at shorter-term shocks that might cause regions to add or subtract to their stocks, we do not model crop storage change over time. This zero or negative CIDR means a region can satisfy its needs from domestic production, which, when enforced in all regions, is expected to lead to decreased participation in global markets (i.e., reduced trade). In contrast, a positive CIDR means a region needs to import cereals to satisfy its domestic cereal consumption. Because every region’s CIDR must be ≤0, no region can have a positive CIDR, so every region’s CIDR equals zero under this constraint.

We enforce this constraint by introducing a cereal production credit created by domestic cereal crop producers. Each unit of domestic production of cereal crops creates one unit of the credit. This credit must be paid for each unit of cereal crop consumed in a region, regardless of whether it is domestically produced or imported. The payment of the credit is returned to the domestic cereal crop producers as a subsidy, which encourages domestic production to meet domestic consumption.


$$cereal\;import\;dependency\;ratio=\frac{cereal\;imports-cereal\;exports}{cereal\;production+cereal\;imports-cereal\;exports}$$ (1)

Given market clearance conditions, where consumption equals to production plus net imports, Eq 1 can be transformed to the following:

$$\begin{array}{l} cereal\;import\;dependency\;ratio=\frac{net\;cereal\;imports}{cereal\;consumption}\\ =\frac{cereal\;consumption-cereal\;production}{cereal\;consumption}\\ =1-\frac{cereal\;production}{cereal\;consumption} \end{array}$$ (2)

The cereal import constraint in GCAM allows us to analyze a range of outcomes relative to the reference (i.e., without this constraint). As GCAM captures economic decisions for various activities across different sectors and regions, we are able to analyze the spatial pattern of model outcomes (e.g., crop land allocation, crop production, global trade, and consumption) under the influence of global zero cereal import dependency and the associated economic changes for producers and consumers in each region. We also explore the implications for irrigation water consumption and crop-based ethanol production (including corn ethanol and sugarcane ethanol) in regions where these are consumed.

Climate change impacts on crop yields are also considered in combination with global zero cereal import dependency. We use the global gridded climate outputs from the Hadley Centre Global Environmental Model [HadGEM; 28, 29] under RCP8.5 and processed them through Persephone [30] to estimate the corresponding crop yield changes under the climate impact for all GCAM crops. Persephone emulates crop yield changes based on yield response sensitivities seen in the Agricultural Model Intercomparison and Improvement Project [AgMIP; 31]. Coordinated Climate-Crop Modeling Project (C3MP) dataset. The climate change impacts on crop yield are incorporated into GCAM as a multiplier to the baseline yield. The corresponding harvest area for each crop is endogenously decided in GCAM, which uses a nested logit architecture in the land-use sector. For more details, please refer to the model documentation.

This one climate scenario under RCP8.5 was selected to understand the underlying mechanism of spatially varying climate change impacts infiltrating across the coupled systems and its interaction with the trade constraints, rather than to quantify a comprehensive range of potential global climate change impacts. Like the global zero cereal import dependency constraint, this should be considered as a thought experiment for exploring the multi-sector dynamics, although our model can be readily applicable to other scenarios.

In total, we explore four model scenarios considering the impact of global zero cereal import dependency and climate change. The four scenarios are:

1. Reference (RF), which is the GCAM baseline model outputs run in 5-year time steps up to 2050;
2. Import Dependency (ID), where zero cereal import dependency constraint is imposed for all GCAM regions globally upon the baseline, starting in 2025;
3. Climate Change (CC), where the climate change impact on crop yield for all GCAM crops across all GCAM regions is imposed upon the baseline beginning in 2020;
4. Import Dependency under Climate Change (IC), which incorporated both ID and CC to study the model responses under the influence of both global zero cereal import dependency and climate change.

GCAM solves for all periods and all scenarios. That is, every market across different scales (e.g., regional market and global market) in the model is solved so that supply and demand are equal.

## 3 Results

### 3.1 Cereal Import Dependency Ratio (CIDR)

**Fig 1:**
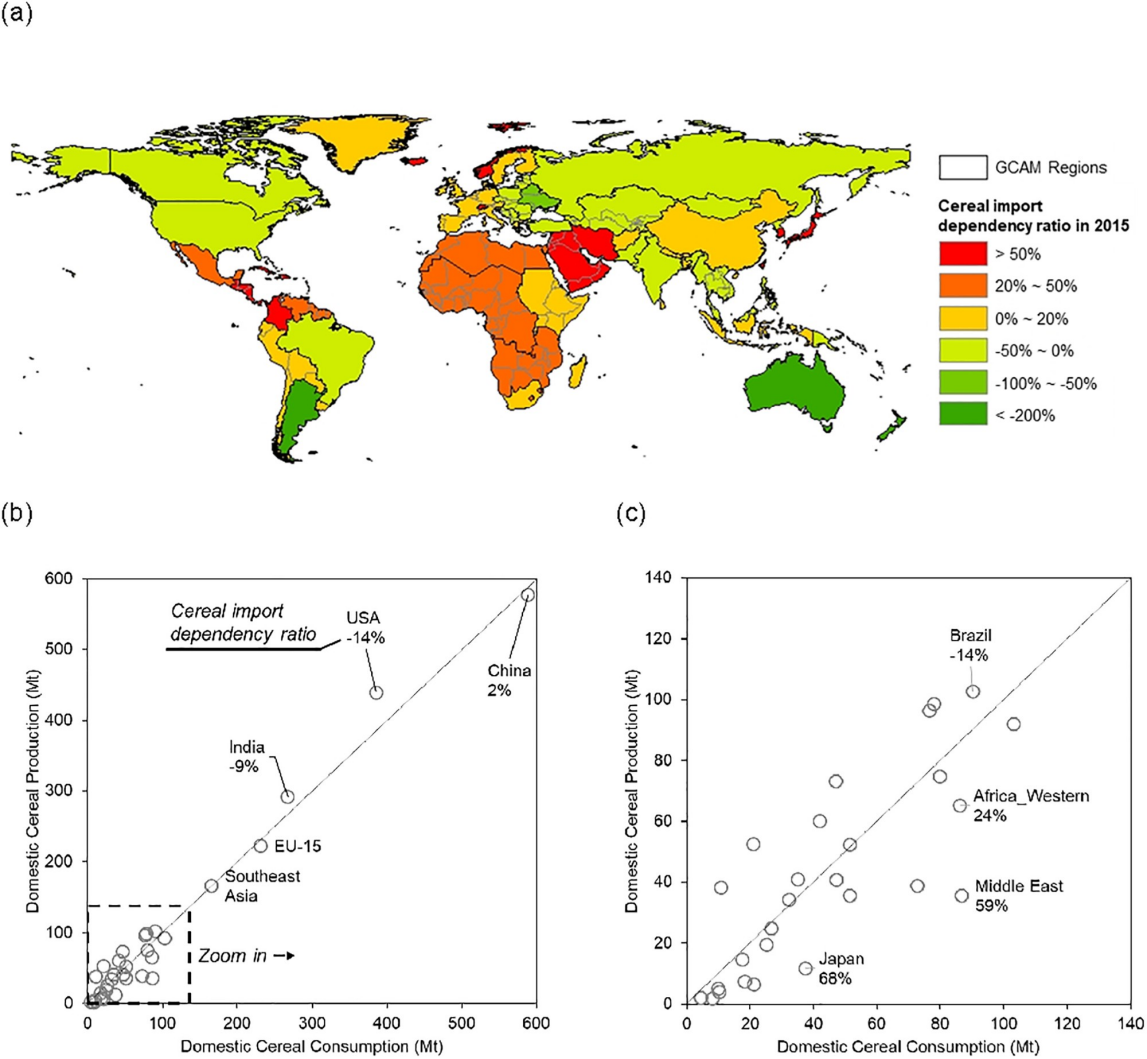
**(a) Global map of cereal import dependency ratio (CIDR)** across GCAM regions in the model base year of 2015, and **(b) the domestic cereal production versus the domestic cereal consumption** in million tons (Mt) in 2015, with a 45-degree line indicating a slope of one (where CIDR equals zero) **(c)** shows a zoom-in portion of (b), as framed in a box.

Fig 1a shows the 2015 (base year) global pattern of CIDR. The CIDR changes between 2015 and 2050 are within ± 14 percentage points for all regions except Brazil and Australia_NZ (a GCAM aggregate region of Australia and New Zealead; S2 Table in S1 File), neither of which depends on cereal imports and have large decreases in their cereal import dependency over the period (S1 Fig in S1 File). In general, regions in South America are projected to have decreasing cereal import dependency over time, while regions in Europe are projected to increase their dependence on imported cereals in 2050, relative to 2015 (S1 Fig in S1 File).

In 2015, the largest cereal consumers are China, USA, and India (Fig 1b). They are also the top cereal producers, with China having the highest CIDR of the three at 2%, and USA and India showing negative CIDR. Regions at the higher end of CIDR include Japan and the Middle East (Fig 1c). A table with more information on major cereal importers and exporters is included in the SI (S3 Table in S1 File).

### 3.2 Global results

**Fig 2:**
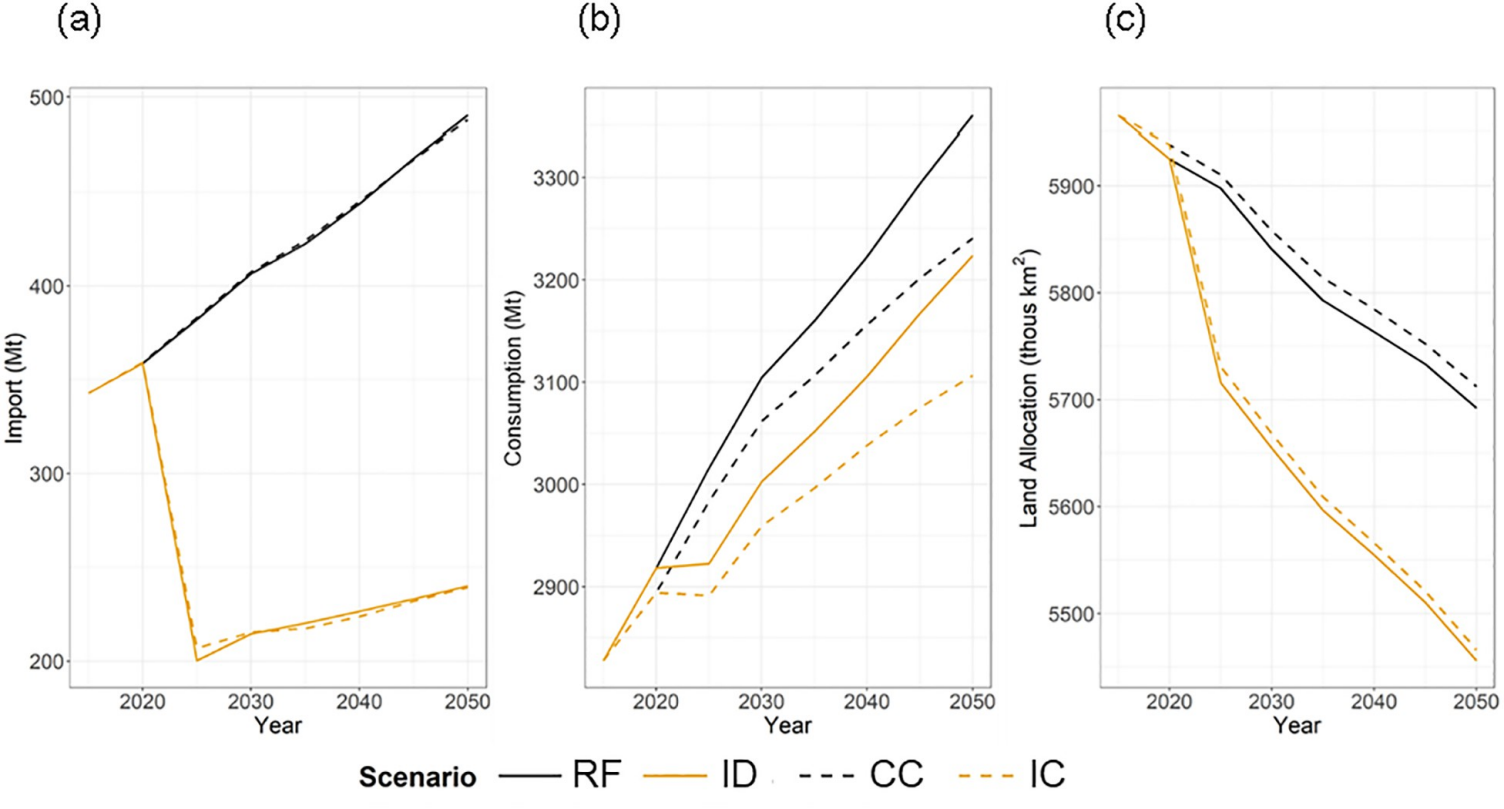
(a) Global total import (equivalent to global total export) of cereals, (b) global total consumption (equivalent to global total production) of cereals, and (c) global total land allocation to cereal crops over 2015–2050 in 5-year time steps under four model scenarios. Note that y-axes are in different scales.

In ID, every region is constrained to cereal import dependency less than or equal to zero beginning in 2025. As a result, no region’s cereal imports exceed exports, with all regions having a CIDR of zero in ID and IC. Global trade of cereals decreases under these scenarios (Fig 2a). In 2025, global total import of cereals in ID and IC decreases to nearly half of the trade quantity in RF, with larger decreases in later periods (Fig 2a). Relative to the effects of ID, the change in global total cereal import quantity due to climate change impacts on agricultural yields, both CC and IC, is very small (Fig 2a).

Despite large decreases in cereal trade in ID, total cereal production and consumption decrease only 3.1%-4.1% over the 2025–2050 period, while climate change accelerates the decrease (Fig 2b). In 2050 the decrease of global cereal consumption in CC is about 3.5% compared to RF, close to the impact of ID. The combined impact of the import dependency constraint and climate change (IC) on global cereal consumption is roughly additive at 7.5%.

The import dependency constraint decreases global land allocated to cereal crops by 3.6% on average (Fig 2c). Climate change impacts on yields are negative at a global level, resulting in increased land allocation to cereal crops in CC and IC, relative to RF and ID, respectively (Fig 2c).

### 3.3 Regional results

To explore the regional differences, we select four representative regions to evaluate the results in more detail: USA, Brazil, Middle East, and Western Africa. USA and Brazil are major cereal exporters in 2015, both with CIDR of -14%, while Middle East and Western Africa are major cereal importers, with CIDR of 59% and 24%, respectively (Fig 1). These regions also represent a wide range of income levels. Climate change has a small effect on CIDR over the projection periods in the four selected regions. In 2050, the CIDR is 2 percentage points higher in CC than RF for USA and Western Africa, and changes minimally for Brazil and Middle East.

#### 3.3.1 Changes of cereal trade, production, and consumption under scenarios

**Fig 3:**
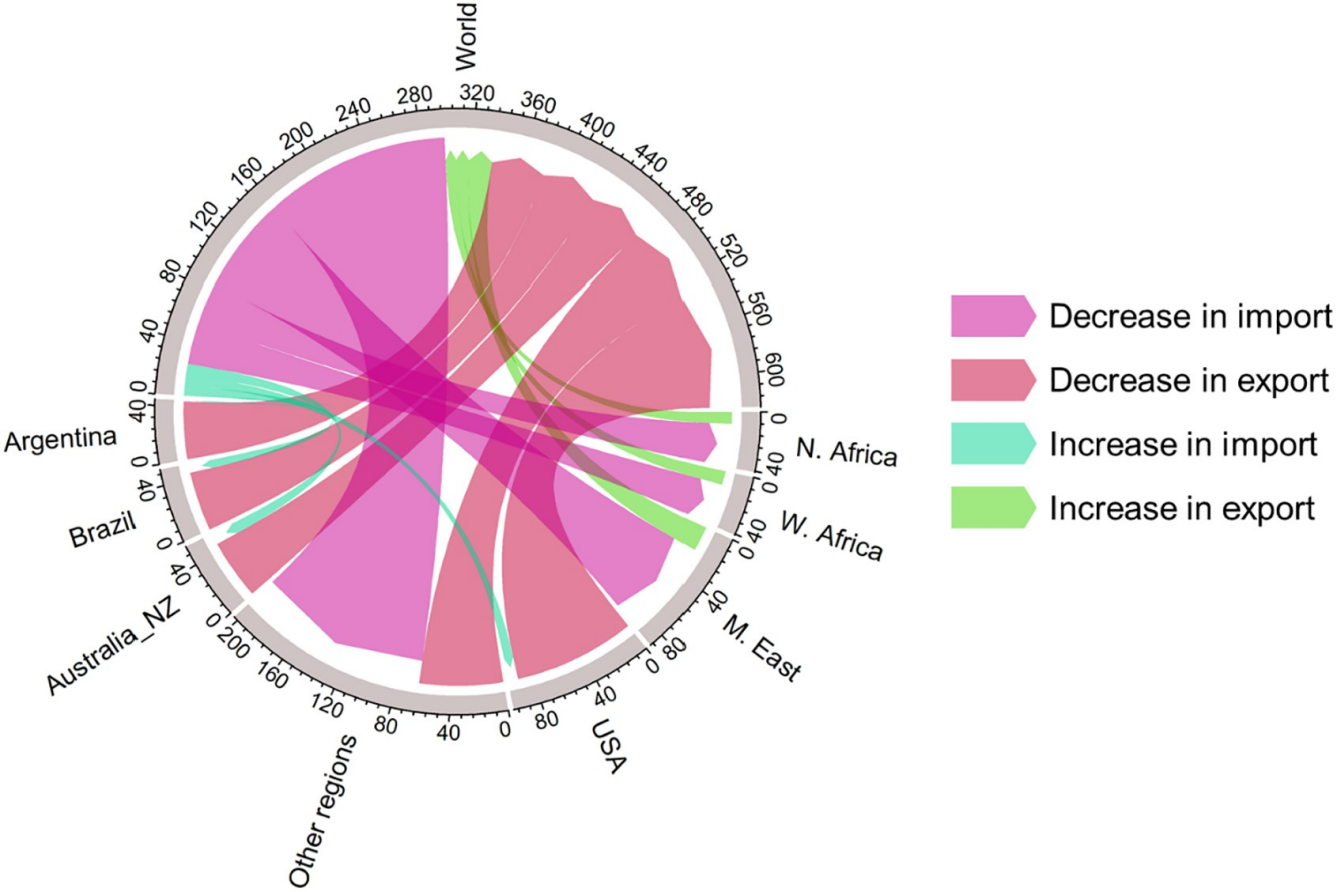
Changes of cereal import and export (Mt) in ID compared to RF in 2050. Note that half of the circle is the global traded market pool, labeled “World”. Arrows that go *to* “World” indicate changes of cereal export *from* regions. Arrows that originate *from* “World” indicate changes of cereal import *to* regions. Top 7 affected regions in terms of changes in cereal trade quantity (Δ import + Δ export) are shown for visualization clarity. The other 25 regions are aggregated into “Other regions”. The color indicates increase or decrease of import or export quantity.

Fig 3 shows regional changes in cereal imports and exports in 2050 in ID compared to RF. Not surprisingly, the regions with the largest changes in trade are those that are the heaviest importers and exporters in RF (S2a Fig in S1 File). For example, USA, Brazil, Argentina, and Australia_NZ, which are the major cereal exporters in RF, show the largest decrease in cereal export in ID due to global trade restrictions. Correspondingly, being the major cereal importers in RF, Middle East, Northern and Western Africa show the largest decrease in cereal import in ID. These patterns are maintained in IC.

**Fig 4:**
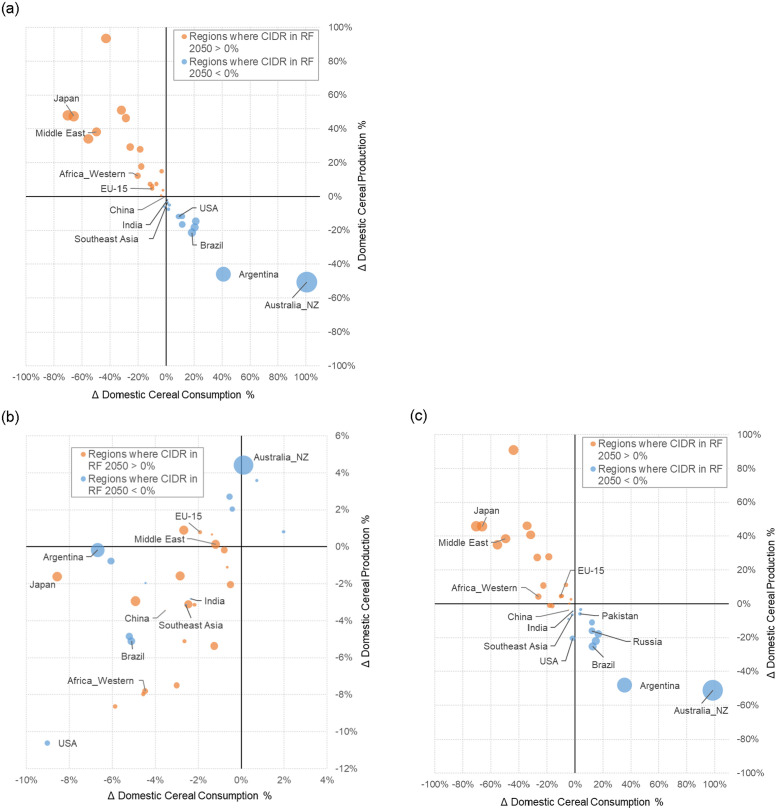
Percent change of domestic cereal production versus percent change of domestic cereal consumption under **(a)** ID, **(b)** CC, and **(c)** IC compared to RF in 2050. The size of the bubble indicates the extent of cereal import dependency ratio (i.e., CIDR) in RF 2050 deviating from 0%. For comparison, the bubble size of Australia_NZ represents a CIDR of -307%; for Japan it is 77%, and close to 0% at the origin such as for China and India, where the CIDR is 1% and -3%, respectively. For all regional CIDR in RF 2050, please refer to S1 Fig in S1 File. Note that the y-axis in CC is on a smaller scale than ID and IC.

**Fig 5:**
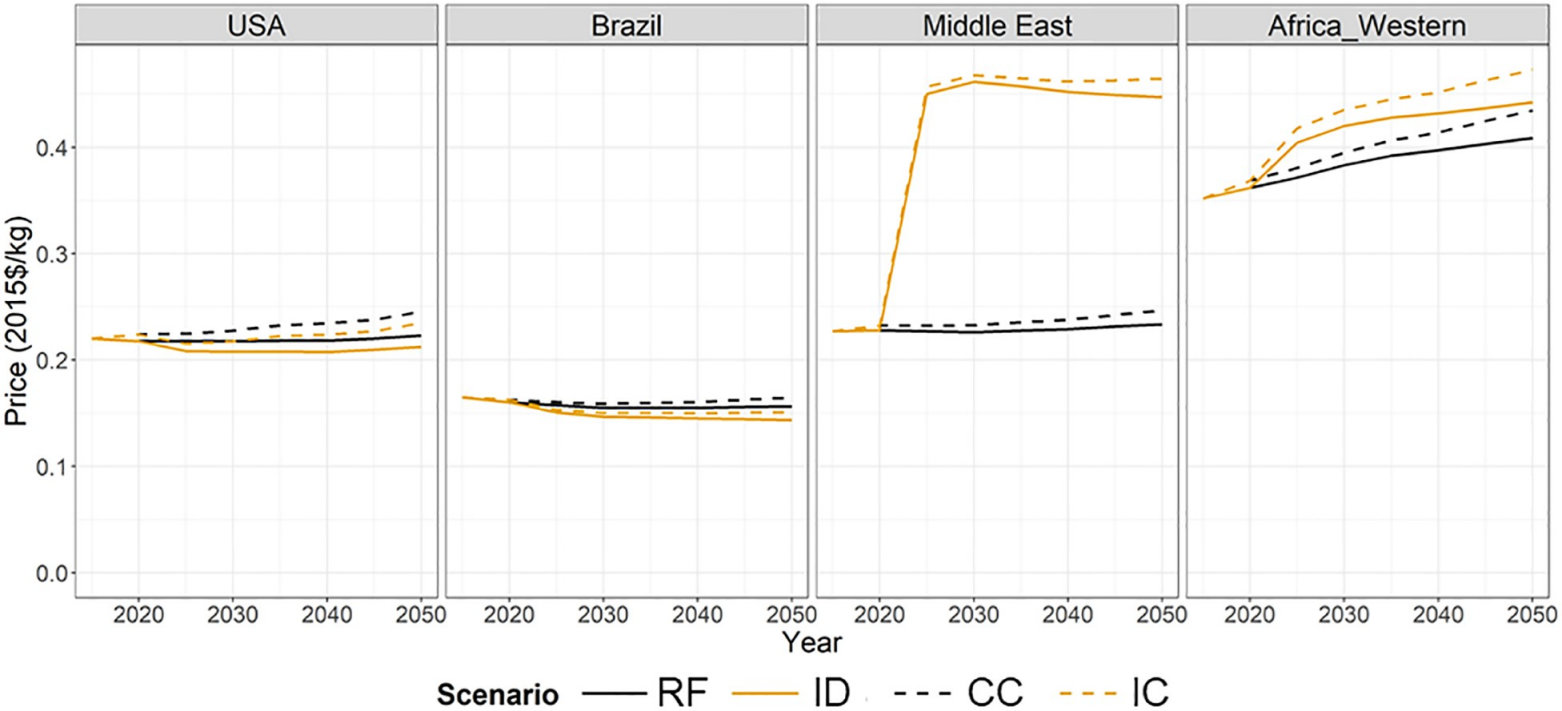
Regional price paid for corn consumption over 2015–2050 in 5-year time steps in selected regions under four model scenarios.

Fig 4 shows the production and consumption patterns that underlie the changes in trade patterns. As expected, regions which have more cereal imports than exports in RF have lower cereal consumption and higher cereal production in ID, and vice versa. That is, cereal importers in RF must increase cereal production in ID, with a resulting increase in price, as on the margin, production in these regions will be more expensive (e.g., Middle East and Western Africa in Fig 5), leading to a decrease in consumption. Cereal exporters in RF decrease production in ID due to lower global demand for their exports, lowering the domestic price of cereals, with a corresponding increase in consumption. These effects are amplified the more CIDR deviates from 0% in RF. For example, Australia_NZ, with a RF CIDR of -307% in 2050, shows changes in domestic cereal production and consumption of approximately -50% and +100%, respectively, in ID in 2050. Comparatively, the USA with a CIDR of -23% shows a -12% and +9% change in cereal production and consumption, respectively. Similarly, cereal importing regions with higher CIDR (>0%) in RF are likely to have a larger reduction in cereal consumption and a greater increase in cereal production in ID. However, both effects are non-linear (Fig 4a) and are influenced by factors such as the land availability, agricultural productivity, ability to substitute cereals for other food commodities, and income levels.

While climate change can influence production and consumption changes in some regions, the impact of the trade perturbations is generally larger (Fig 4a and 4b). One exception is the USA, for which the changes for production and consumption in 2050 in CC, -10.6% and -9.0%, respectively, are comparable in magnitude to the production and consumption changes in ID, -11.8% and +8.9%, respectively. These changes are approximately additive in IC, where the increase in cereal consumption in ID is offset by the decrease in CC, resulting in a consumption change of -1.8% in IC (Fig 4c). Additional climate change impacts on crop production are included in SI (S3 Fig in S1 File).

#### 3.3.2 Cereal prices under scenarios

Regional prices of cereal crops, defined as the weighted average of domestic and imported prices including the subsidy price (cereal domestic production credit price paid for consumption), rise in regions with RF CIDR>0% (Western Africa, Middle East) and fall in regions with RF CIDR<0% (USA, Brazil) under the effects of global zero cereal import dependency in ID (Fig 5). In regions with RF CIDR>0%, production expands into land in which it is more costly to produce, increasing prices in ID, while regions which do not depend on cereal imports in RF will have lower production and contraction of land in ID (S4 Fig in S1 File), resulting in reduced prices.

In high-importing regions, producers need higher domestic prices to stimulate domestic cereal production in ID (e.g., Middle East and Western Africa in Fig 5) and, with the import dependency constraint, net importers do not benefit from the comparatively low price of cereal crops in the global market. Fig 5 shows these effects on the regional corn prices in the four representative regions. The effects of the import dependency constraint on cereal prices depend on how much and in which direction the CIDR in RF deviates from 0%. USA and Brazil, both with RF CIDR of -14% in the base year (Fig 1), show a slight decrease in regional prices in ID compared to RF (Fig 5). Western Africa, with RF CIDR of 24%, shows a slight increase in the regional prices, while the Middle East, with RF CIDR of 59%, shows a larger increase in regional prices than Western Africa.

The regional price of corn is higher for these four regions in CC than RF and in IC than ID (Fig 5). In the USA and Brazil, the increase in price due to climate change impacts on crop yields is comparable to the decrease in price due to the import dependency constraint, thus price in IC is similar to RF. In Western Africa and Middle East, with CIDR>0%, the effects on prices from the import dependency constraint are much larger than from climate change.

The cereal domestic production credit is modeled as a subsidy to the producers to increase production sufficiently to meet domestic demand. So the price received by producers includes both the “market” component of the price and a subsidy from the cereal domestic production credit (S5 Fig in S1 File). Under ID, the price received by producers in the net exporting regions, USA and Brazil, is slightly lower than RF. For the net cereal importers in RF, the total price paid to producers, including subsidy and market price, is higher than in RF; for the Middle East, the price received by producers is nearly twice as large in ID than in RF.

#### 3.3.3 Impacts on non-cereal crops and other key outcomes

We also explore the effects of global zero cereal import dependency on other key outcomes: non-cereal crop production, consumption, exports, and imports; crop producer revenue; net trade revenue; caloric consumption; the food expenditure share of income; irrigation water consumption; and ethanol production using corn and sugarcane. Tables 1 and 2 show the 2050 results for USA and Middle East—the top net exporter and importer of cereals in RF (See S1 File for Western Africa and Brazil results). Note that the Middle East region defined here covers not just large oil-producers but spans diverse countries from the eastern Mediterranean to the Persian Gulf (S2 Table in S1 File). Also, among the four regions, only Brazil has sugarcane ethanol production, while the three others have corn ethanol production, a cereal crop-based ethanol that is more directly affected by the cereal trade constraints. Although sugarcane is not a cereal crop, sugarcane ethanol production and consumption are indirectly impacted by the global zero cereal import dependency. For discussion on the results of sugarcane ethanol across scenarios, please refer to SI. Ethanol results (both corn and sugarcane) across all GCAM regions are also shown in S7 and S8 Figs in S1 File. The following results are based on Tables 1 and 2 for the USA and Middle East, respectively.

**Table 1:** Summary of key variable results in 2050 for the USA. The numbers in the parentheses indicate the percent change compared to RF. Numbers are rounded. Mt—Million tons.

Key Variable	*of*	RF	ID	CC	IC
**Import dependency ratio**	*Cereals*	-23%	0%	-21%	0%
**Production (Mt)**	*Cereals*	523	461 (-11.8%)	467 (-10.6%)	416 (-20.4%)
	*Non-Cereals*	348	346 (-0.6%)	343 (-1.5%)	340 (-2.3%)
	*All Crops*	871	808 (-7.3%)	811 (-7.0%)	756 (-13.2%)
**Consumption (Mt)**	*Cereals*	424	461 (8.9%)	385 (-9.0%)	416 (-1.8%)
	*Non-Cereals*	324	306 (-5.2%)	340 (4.9%)	324 (0.0%)
	*All Crops*	747	767 (2.8%)	726 (-3.0%)	740 (-1.0%)
**Exports (Mt)**	*Cereals*	113	22 (-80.9%)	98 (-13.3%)	22 (-80.8%)
	*Non-Cereals*	110	123 (11.9%)	97 (-11.7%)	109 (-1.5%)
	*All Crops*	224	145 (-35.2%)	196 (-12.5%)	130 (-41.7%)
**Imports (Mt)**	*Cereals*	14	22 (53.7%)	16 (15.6%)	22 (54.7%)
	*Non-Cereals*	86	83 (-3.0%)	94 (10.4%)	92 (7.6%)
	*All Crops*	100	105 (5.0%)	111 (11.1%)	114 (14.2%)
**Net Trade Revenue (billion 2015$)**	*Cereals*	31.5	1.75 (-94.5%)	27.9 (-11.2%)	1.71 (-94.6%)
	*Non-Cereals*	12.7	19.8 (55.5%)	3.6 (-71.8%)	10.3 (-19.4%)
	*All Crops*	44.2	21.5 (-51.3%)	31.5 (-28.7%)	12.0 (-72.9%)
**Crop Producers’ Revenue (billion 2015$)**	*Cereals*	155	128 (-17.8%)	150 (-3.4%)	125 (-19.3%)
	*Non-Cereals*	200	198 (-1.1%)	208 (3.9%)	205 (2.4%)
	*All Crops*	356	326 (-8.4%)	358 (0.7%)	331 (-7.1%)
**Caloric Consumption (Kcal/cap/day)**	*Cereals*	1048	1075 (2.5%)	1044 (-0.4%)	1070 (2.1%)
	*Non-Cereals*	2701	2682 (-0.7%)	2687 (-0.5%)	2668 (-1.2%)
	*Staples* *	1142	1142 (0.0%)	1141 (0.0%)	1141 (0.0%)
	*All Food*	3749	3756 (0.2%)	3731 (-0.5%)	3738 (-0.3%)
**Food Expenditures as a share of GDP**	*All Food*	0.31%	0.30%	0.34%	0.33%
**Irrigation Water Consumption (km** ^**3**^ **)**	*Cereals*	42.4	36.2 (-14.5%)	37.7 (-11.1%)	32.5 (-23.3%)
	*Non-Cereals*	37.0	36.7 (-0.7%)	41.3 (11.7%)	41.0 (10.9%)
	*All Crops*	79.4	72.9 (-8.1%)	79.0 (-0.5%)	73.5 (-7.4%)
**Ethanol Production (EJ)**	*Corn*	1.28	1.42 (10.4%)	1.05 (-18.1%)	1.15 (-10.0%)
**Corn Consumption for Ethanol (Mt)**		143	158 (10.4%)	117 (-18.1%)	129 (-10.0%)
**Share of Corn Consumption for Ethanol**		41.0%	42.5%	38.4%	39.9%

*Staples include cereals as well as roots and tubers in our model.

**Table 2:** Summary of key variable results in 2050 for the Middle East. The numbers in the parentheses indicate the percent change compared to RF. Numbers are rounded. Mt—Million tons.

Key Variable	*of*	RF	ID	CC	IC
**Import dependency ratio**	*Cereals*	64%	0%	63%	0%
**Production (Mt)**	*Cereals*	52	72 (38.3%)	52 (0.1%)	72 (38.5%)
	*Non-Cereals*	147	169 (15.1%)	151 (3.1%)	174 (18.7%)
	*All Crops*	198	240 (21.1%)	203 (2.3%)	246 (23.8%)
**Consumption (Mt)**	*Cereals*	142	72 (-49.6%)	140 (-1.2%)	72 (-49.5%)
	*Non-Cereals*	197	252 (27.4%)	199 (1.2%)	255 (28.9%)
	*All Crops*	339	323 (-4.6%)	339 (0.2%)	326 (-3.7%)
**Exports (Mt)**	*Cereals*	0.7	19.4 (2599.0%)	0.8 (6.2%)	19.7 (2640.3%)
	*Non-Cereals*	5.3	4.3 (-18.6%)	5.6 (6.0%)	4.5 (-15.0%)
	*All Crops*	6.0	23.7 (294.7%)	6.4 (6.0%)	24.2 (302.8%)
**Imports (Mt)**	*Cereals*	90.8	19.4 (-78.6%)	89.1 (-1.9%)	19.7 (-78.3%)
	*Non-Cereals*	55.4	87.0 (57.0%)	53.6 (-3.2%)	84.8 (53.2%)
	*All Crops*	146.2	106.4 (-27.2%)	142.7 (-2.4%)	104.6 (-28.5%)
**Net Trade Revenue (billion 2015$)**	*Cereals*	-25.6	-1.4 (-94.5%)	-25.5 (-0.4%)	-1.5 (-94.3%)
	*Non-Cereals*	-15.0	-22.3 (49.0%)	-15.3 (2.4%)	-22.9 (53.1%)
	*All Crops*	-40.6	-23.7 (-41.6%)	-40.8 (0.6%)	-24.4 (-40.0%)
**Crop producers’ Revenue (billion 2015$)**	*Cereals*	26	44 (71.9%)	26 (0.5%)	45 (73.3%)
	*Non-Cereals*	118	133 (12.6%)	122 (3.7%)	138 (16.8%)
	*All Crops*	144	177 (23.2%)	148 (3.1%)	183 (27.0%)
**Caloric Consumption (Kcal/cap/day)**	*Cereals*	1182	1078 (-8.7%)	1181 (0.0%)	1076 (-8.9%)
	*Non-Cereals*	1477	1585 (7.3%)	1473 (-0.3%)	1582 (7.2%)
	*Staples* *	1230	1230 (0.0%)	1230 (0.0%)	1230 (0.0%)
	*All Food*	2659	2663 (0.2%)	2654 (-0.2%)	2659 (0.0%)
**Food Expenditures as a share of GDP**	*All Food*	0.82%	0.85%	0.84%	0.88%
**Irrigation Water Consumption (km** ^**3**^ **)**	*Cereals*	62.3	86.8 (39.3%)	61.6 (-1.1%)	86.1 (38.2%)
	*Non-Cereals*	99.9	109.5 (9.6%)	101.8 (1.9%)	111.7 (11.8%)
	*All Crops*	162.2	196.3 (21.0%)	163.4 (0.7%)	197.8 (22.0%)
**Ethanol Production (EJ)**	*Corn*	0.181	0.007 (-96.0%)	0.156 (-13.8%)	0.006 (-96.8%)
**Corn Consumption for Ethanol (Mt)**		20	0.8 (-96.0%)	17.4 (-13.8%)	0.6 (-96.8%)
**Share of Corn Consumption for Ethanol**		55.2%	14.1%	54.1%	12.1%

*Staples include cereals as well as roots and tubers in our model.

*Non-cereal crop production*, *consumption*, *and trade*. The effect of ID on USA non-cereal production is also negative as the effect on USA cereal production, though very small (<0.6%). This is due to two competing drivers. First, the relatively low cereal prices lead to an increase in domestic consumption of cereals and hence a decrease in domestic consumption of non-cereals. Second, as importing regions are forced to increase imports of non-cereal crops displacing cereal imports, exporting regions increase their non-cereal crop export. As a result, the combined effect on non-cereal production is small. The effects of this constraint on non-cereal crops are observed in the Middle East, which increases production, consumption, and imports of non-cereal crops, to offset the 5% decrease in consumption of cereals in ID.

In CC, the USA generally decreases non-cereal crop production, exports, and imports. However, consumption of non-cereal crops increases, partially offsetting the decrease in cereal consumption. In the Middle East, yields for non-cereal crops generally increase in CC, leading to an increase in production and consumption and a decrease in imports.

In IC, the effects of trade perturbations dominate in the Middle East with production, consumption, and trade changes for non-cereals resembling those in ID. In the USA, however, the CC impacts are slightly stronger than ID in these outcomes for non-cereal crops.

The combined effects of changes in quantity and price will impact other measures of well-being, including trade revenue, producer’s revenue, and consumer’s food expenditure. The direction of these impacts, and the extent to which climate change or trade perturbations impact regions are dependent on whether regions are net importers or exporters and how the yields of their major crops are expected to be affected by climate change.

*Net trade revenue and producer revenue*. As a major cereal exporter in RF, USA experiences a decrease in net export revenue from cereals in ID, while increasing export revenue from non-cereal crops. The net effect of ID on total crop export revenue is negative, resulting in a roughly 50% loss relative to RF. In CC, trade revenue decreases for both cereal and non-cereal crops, but the total loss, roughly 30%, is smaller than that in ID. The combined effects in IC are large, with a loss in trade revenue of nearly 75%.

In ID, the Middle East shows a 94% reduction in cereal net import expenditures. However, these are partially offset by increased trade expenditures for non-cereal crops, with a total reduction in crop trade expenditures of 42%. In CC, the revenue changes are very small, with slightly increased crop trade expenditures. In IC, changes in trade revenue are dominated by the effects of the cereal import dependency constraints.

The effects of the import dependency constraint on USA producer revenue from cereals (-18%) are directionally the same as trade revenue (-94%), though only one-fifth as large. While non-cereal trade revenue increases in ID, producer revenue from no-cereal crops has a slight decrease. In CC, USA cereal producer revenue decreases slightly, while total producer revenue increases slightly, driven by increased prices for non-cereal crops. As with trade revenues, the results in IC are similar to ID, as the trade perturbations is the dominant effect. In the Middle East, producer revenues from cereal crops increase in ID, CC, and IC, where revenues also increase for non-cereal crop producers.

*Caloric consumption and food expenditure*. We also show caloric consumption from cereal, non-cereal, and staple foods, a category that includes cereals as well as roots and tubers. The category of staple foods provides relatively inexpensive, but often less nutritious calories than non-staples. The overall effects of trade perturbations on staple caloric consumption are small, less than 0.5% for both regions and all scenarios, where changes in cereal caloric consumption are offset by changes in other staple foods, reaching a net zero change in staple consumption. However, changes in prices also result in changes in non-staple consumption. The Middle East has reductions in total calories in CC, driven by reductions in non-staple consumption. The USA has a slight increase in total caloric consumption in ID, but decreases in CC and IC.

Like producers, consumers are affected by the net impact of changes in both quantity and price. One measure of well-being is the food expenditure share of income (measured as regional crop prices times food consumption, as a share of GDP). In the Middle East the food expenditure share increases due to both the trade perturbations and climate change, while in the USA it decreases in ID but increases in CC and IC. Overall, consumers in most cereal importing regions (in RF) are paying more for food in all scenarios than in RF. In scenarios where crop producer revenues decrease while food expenditures increase, the producers, who are also food consumers, would be financially worse off. However, in scenarios where crop producers’ revenues increase, the net effect is ambiguous for producers, as they have both higher revenues and higher food expenditures.

*Water consumption*. In the water sector, the distribution of irrigation water consumption changes significantly, decreasing roughly 8% in the USA and increasing 21% in the Middle East due to the impact of cereal import dependency constraints. In the USA, irrigation water for non-cereal crops decreases slightly, associated with the decrease in non-cereal crop production in ID. In the Middle East, irrigation water consumption for both cereal and non-cereal crops increases, due to the need to intensify production in ID. In CC, irrigation water consumption decreases for cereal crops and increases for non-cereal crops in both the USA and Brazil, although the magnitude of change is much smaller in the Middle East than USA. Additionally, in the Middle East, the changes in irrigation water consumption in CC is much smaller than in ID, as international trade is able to mitigate some of the negative impacts on crop yields from climate change. Therefore, in IC, the change of irrigation water consumption is similar to ID given its dominant effects in the Middle East. In contrast, in the USA, the increase in irrigation water for non-cereal crops in CC is much larger in magnitude than the decrease in ID, resulting in an increase in irrigation water for non-cereal crops in IC. Results for irrigation water consumption across all regions are shown in S6 Fig in S1 File.

*Ethanol production*. While CC has a similar percent change impact on corn ethanol production in the Middle East and USA, -14% and -18%, respectively, ID results in very different impacts, with a 96% decrease in Middle East and an 11% increase in USA. Note, though, that the corn ethanol production in the Middle East in 2050 is very small in RF, with <15% of the corn ethanol production of the USA. However, the magnitude of change in corn ethanol production in the Middle East in ID relative to RF is greater than the change in USA (Tables 1 and 2). The near complete elimination of corn ethanol in the Middle East in ID is due to the regional scarcity of cereal crops and a more elastic demand for ethanol than food. In contrast, in the USA, a major corn exporter, corn ethanol becomes relatively more competitive compared to other liquid fuels domestically due to the decrease in global demand for corn in ID. In general, in regions where corn ethanol is available, cereal net importers in RF reduce corn ethanol production in ID while the cereal net exporters increase corn ethanol production (S7 Fig in S1 File).

## 4 Discussion

Global human and physical Earth systems interact with each other through multiple interconnected systems. Perturbations to any of these systems carry implications for both human and physical Earth systems everywhere. Understanding these systems and their interconnectivity can be difficult because only the total effects of multiple, simultaneous perturbations and responses are observed. For instance, the global food supply chain was disrupted by the 2007–08 international food crisis due to extreme weather impacts on crop yields and the COVID-19 pandemic. A recent study showed that the COVID-19 outbreak led to drastic reduction in trade interconnectedness among countries [32]. The long-term effects of such disruptions on agricultural trade are uncertain.

To better understand how human and physical Earth systems interact around a changing climate and constraints on global trade, we evaluate the individual and combined impacts of these changes on multiple sectoral outcomes through 2050. While we analyze only one realization of climate change impacts on crop yields and one alternative trade perturbation scenario using global cereal import dependency constraints, the insights to the relative impacts across regions are likely similar to alternate impacts, though the magnitude may differ depending on estimates of yield impacts and degrees of import dependency constraints. The yield changes used in this study have also been applied in other studies [17, 33, 34], with some crops and regions experiencing increases in yields under climate change, but overall climate impacts on agricultural yields are negative. Changes to the land, bioenergy, and water systems would vary in the real world, depending on the degree to which countries might impose constraints on import dependency to combat uncertainty from supply chain disruptions and the regional climate change impacts on crop yields. However, the objective of this study is not to predict future climate impacts or trade patterns, but rather to explore how outcomes across multiple systems could be affected by changes in trade patterns and how those results could be mitigated or amplified with the effects on crop yields due to a simultaneously changing climate.

Our findings show that the global trade quantity of cereals decreases to almost half of the baseline quantity under the effects of global zero cereal import dependency across all regions, with much lower decreases in cereal production and consumption. Although the overall impact of this constraint is larger than climate change on cereal production and consumption, climate change has an important role in some regions such as the USA. The import dependency constraints for cereals will also affect production, consumption, and trade of non-cereal crops indirectly.

Reducing import dependence will reduce regions’ domestic agriculture system sensitivity to fluctuations in the international agricultural supply chains. However, such changes increase the countries’ susceptibility to domestic shocks, such as drought or floods. These constraints also have the potential to negatively impact measures of economic well-being, including trade and producer revenues as well as food expenditures. Changes in domestic production due to import dependency constraints will also impact the level of agricultural inputs, such as land and water, potentially accelerating the scarcity in regional resources. Climate change impacts on agricultural yields will also influence these measures. In some cases, climate change and import dependency constraints slightly amplify each other, as in total irrigation water consumption in the Middle East, which increases by 21% and 0.7% under ID and CC, respectively, but 22% under IC.

In addition to the implications of the trade perturbations and climate change to agriculture, bioenergy, and water outcomes, there may be other potential far-reaching multi-sector outcomes that are not evaluated directly here and could be of interest in future studies. For example, the effects on ecosystems are not included in this study, while other studies have shown that agricultural trade may be positively correlated with deforestation [35–37]. This could also have an impact on ecosystem carbon storage, net emissions, and biodiversity [38]. For another example, industries involved in the production and distribution of agricultural inputs, such as seeds, fertilizers, pesticides, and machinery, may experience changes in demand and profits if farmers modify their crop choices or adjust their production practices in response to the trade perturbations and climate change. As shown by a previous study, international agricultural trade can significantly redistribute the global phosphorus (P) cycle, particularly due to consumption of P fertilizers [39]. A third example is the effect on the overall energy market and alternative energy investment options due to the fluctuations in biofuel production, a potentially important energy source in the future [40–42].

Synergies and tradeoffs were revealed as we systematically alter trade patterns and climate. While reducing import dependency could potentially lower the risk in situations of supply chain disruptions, other region-specific costs and benefits differ across sectoral outcomes and the effects of climate change alter the relative changes. Further, in the real world, the relative impact of trade perturbations and climate change may differ depending on specific constraints and effects of climate change that are not modeled here. For instance, regional import dependency constraints may not be as extreme as those modeled in this study, where different regions would likely implement varying levels of import dependency extents for varying baskets of different agricultural commodities. The climate change impacts used here represent long-term averages of climate impacts on crop yields under a single climate model’s representation of future change. The effects of inter-annual variability in weather shocks, such as droughts or floods, are likely to have sharper impacts on regional production than long-term climate averages, potentially changing the relative effects of disruption from supply chains compared to the disruption from domestic production losses. Thus, the real-world implications will depend on which and to what degree countries act to reduce import dependency to combat uncertainty from supply chain disruptions and the local effects of climate change.

## Supporting information

S1 File Supporting information for agriculture, bioenergy, and water implications of constrained cereal trade and climate change impacts. This file contains figures and tables cited in the main text.
(DOCX)
