# Supplementary material for: Agriculture, bioenergy, and water implications of constrained cereal trade and climate change impacts
Source: PLoS One. 2023 Sep 15;18(9):e0291577. doi: 10.1371/journal.pone.0291577 (PMC10503731; doi:10.1371/journal.pone.0291577)
Supplement: S1 File — This file contains figures and tables cited in the main text. (DOCX) [file pone.0291577.s001.docx]

Supporting Information for

**Agriculture, bioenergy, and water implications of constrained cereal trade and climate change impacts**

Ying Zhang^1*^, Stephanie Waldhoff^1^, Marshall Wise^1^, Jae Edmonds^1^, and Pralit Patel^1^

^1^Joint Global Change Research Institute, Pacific Northwest National Laboratory, College Park, MD, USA.

Corresponding author: Ying Zhang (ying.zhang@pnnl.gov)

*https://orcid.org/0000-0002-0036-7502

**Introduction**

Please refer to the main text, where the SI figures and tables are cited.

(a)

(b)


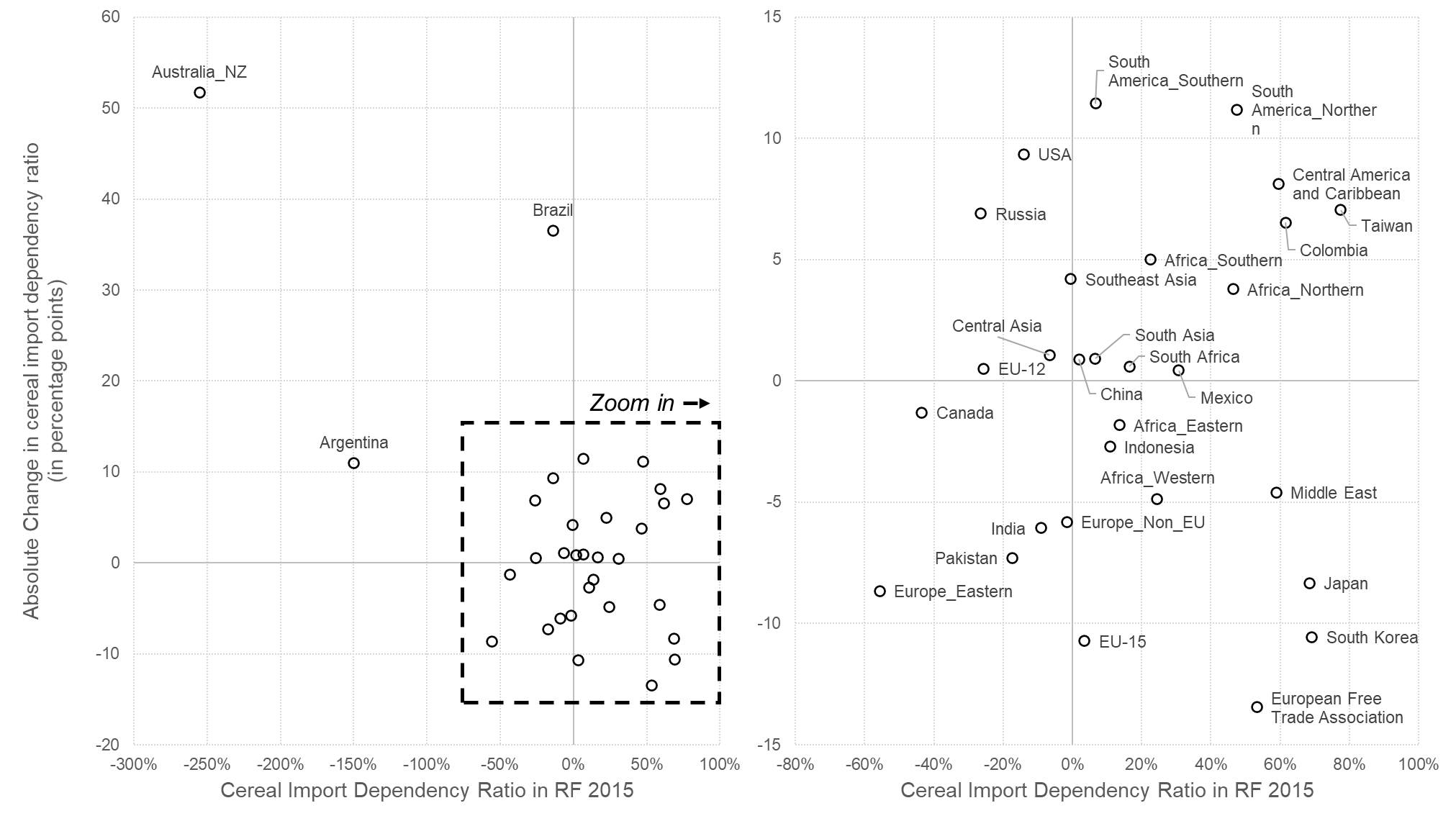


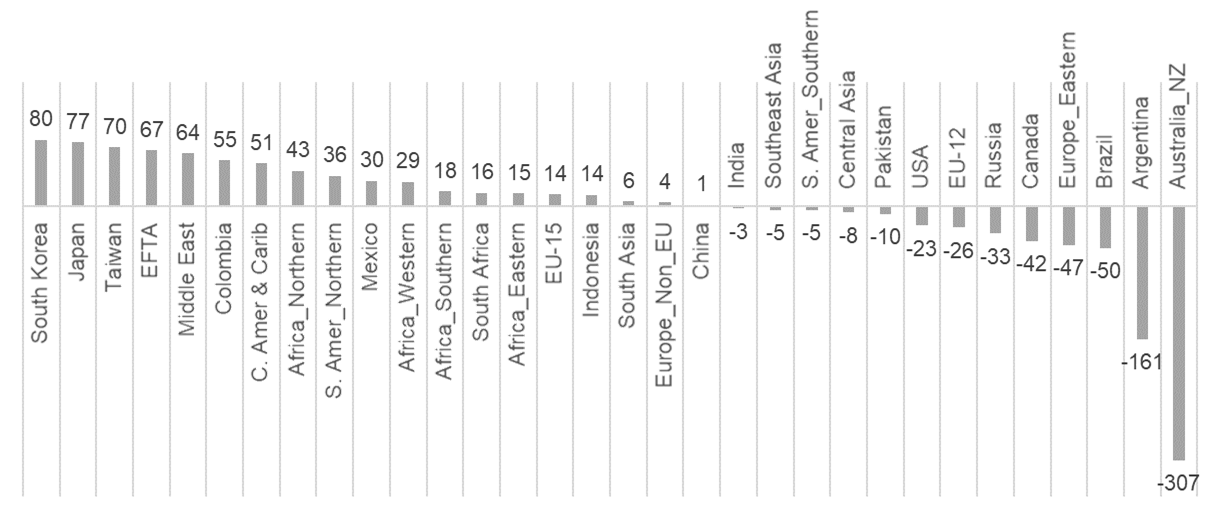


(c) Cereal Import Dependency Ratio (CIDR) in 2050 RF

**S1 Fig. (a) Absolute change of cereal import dependency ratio** (in percentage points) in 2050 relative to the value in 2015, for all GCAM regions in RF. (b) zooms in the dashed box in (a). (c) shows the **regional cereal import dependency ratio** in 2050 RF, ranked from the largest to the smallest. Note that “EFTA” is short for “European Free Trade Association”; “C. Amer & Carib” is short for “Central America and Caribbean”; “S. Amer" is short for “South America”.


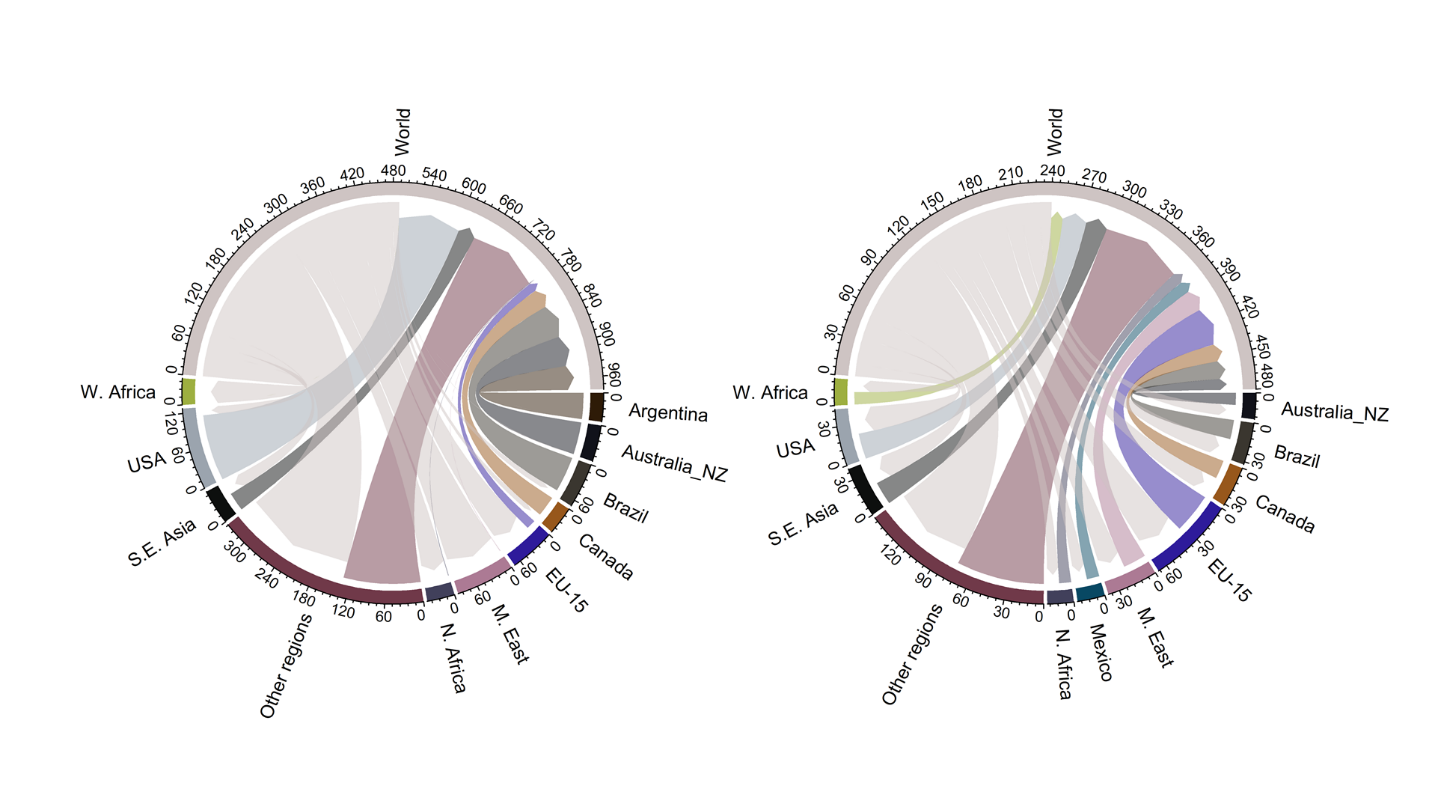


(b)

(a)

**S2 Fig. Import and export of cereal crops in total** (Mt) across regions in 2050 under the (a) RF and (b) ID scenario. Note that half of the circle is the global traded market pool, labeled “World”. Arrows that go *into* “World” indicate export from regions differentiated by colors. Arrows that originate *from* “World” indicate import to regions. Top 10 players in terms of trade quantity of cereal total (import + export) are shown. The rest are aggregated into “Other regions”. Note that the scales are different.


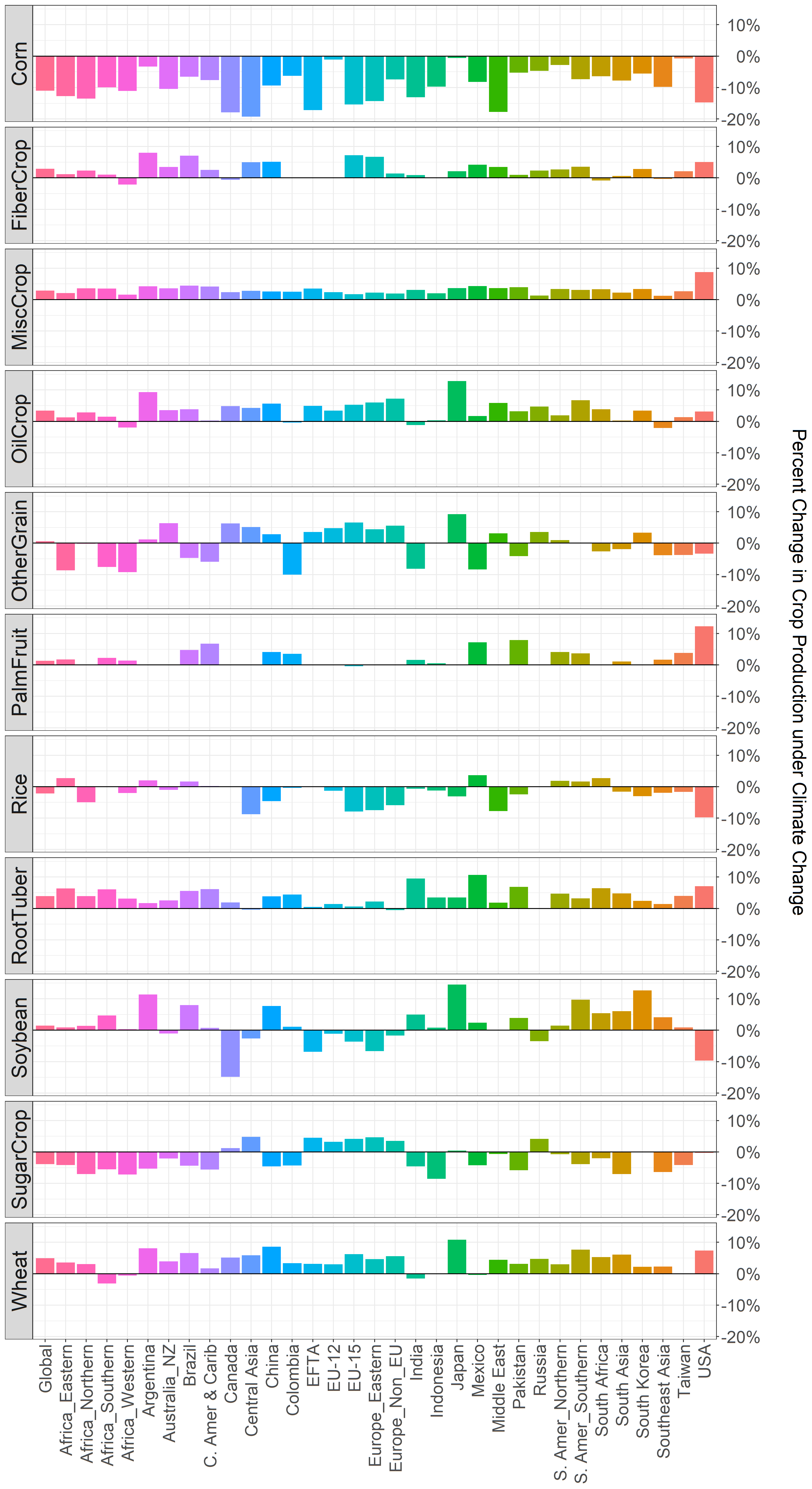


**S3 Fig. Percent change of crop production** (Mt) in 2050 CC compared to the value in 2050 RF. Note that in the x-axis labels “EFTA” is short for “European Free Trade Association”; “C. Amer & Carib” is short for “Central America and Caribbean”; “S. Amer" is short for “South America”.


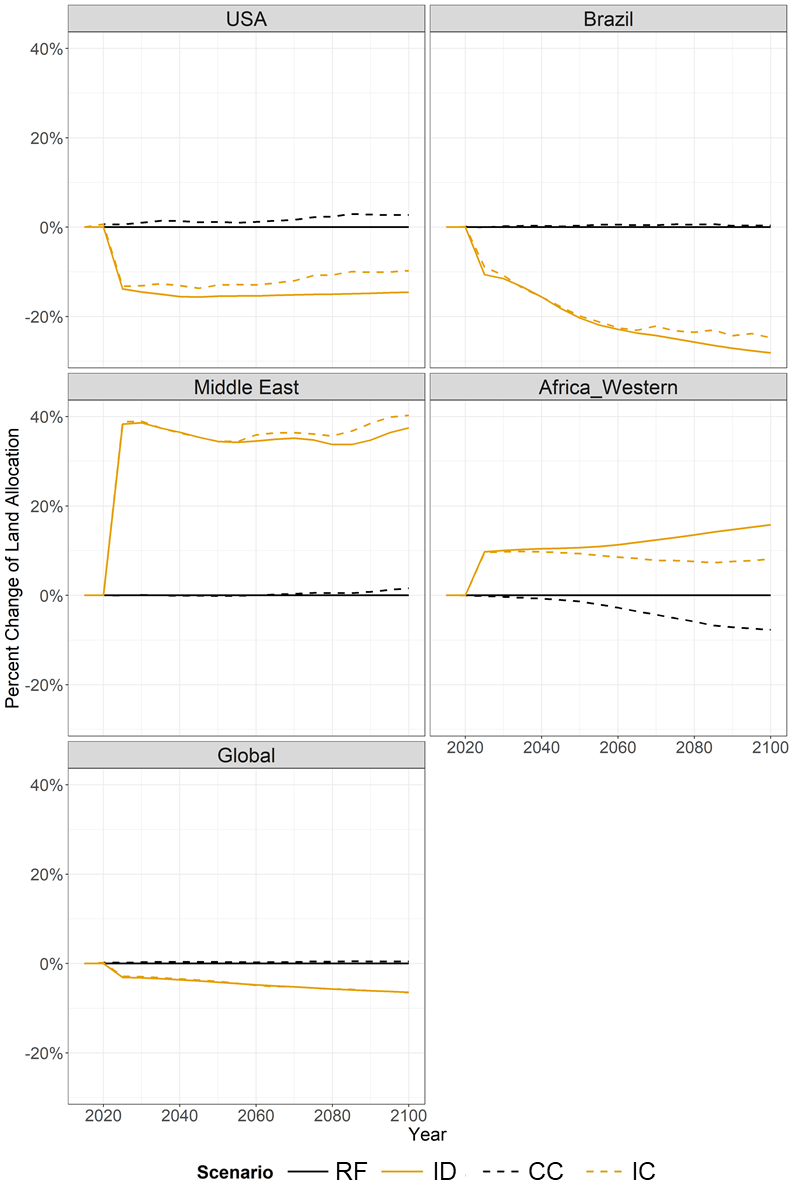


**S4 Fig. Percent change of land allocation to cereals** in selected regions and globally under four model scenarios, compared to the RF.


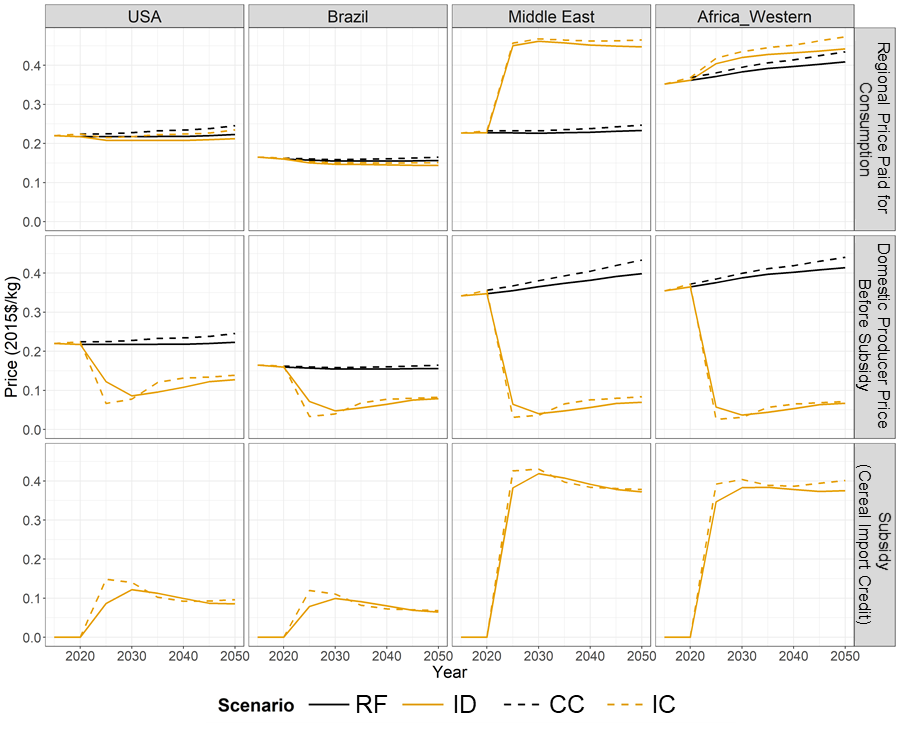


**S5 Fig. Regional price paid for consumption** (first row), **domestic producer price excluding the cereal import credit** (second row), and **the price of the cereal import credit** (paid to the crop producers as a subsidy under the ID and IC scenario; third row) over 2015-2050 in 5-year time steps in selected regions under four model scenarios.


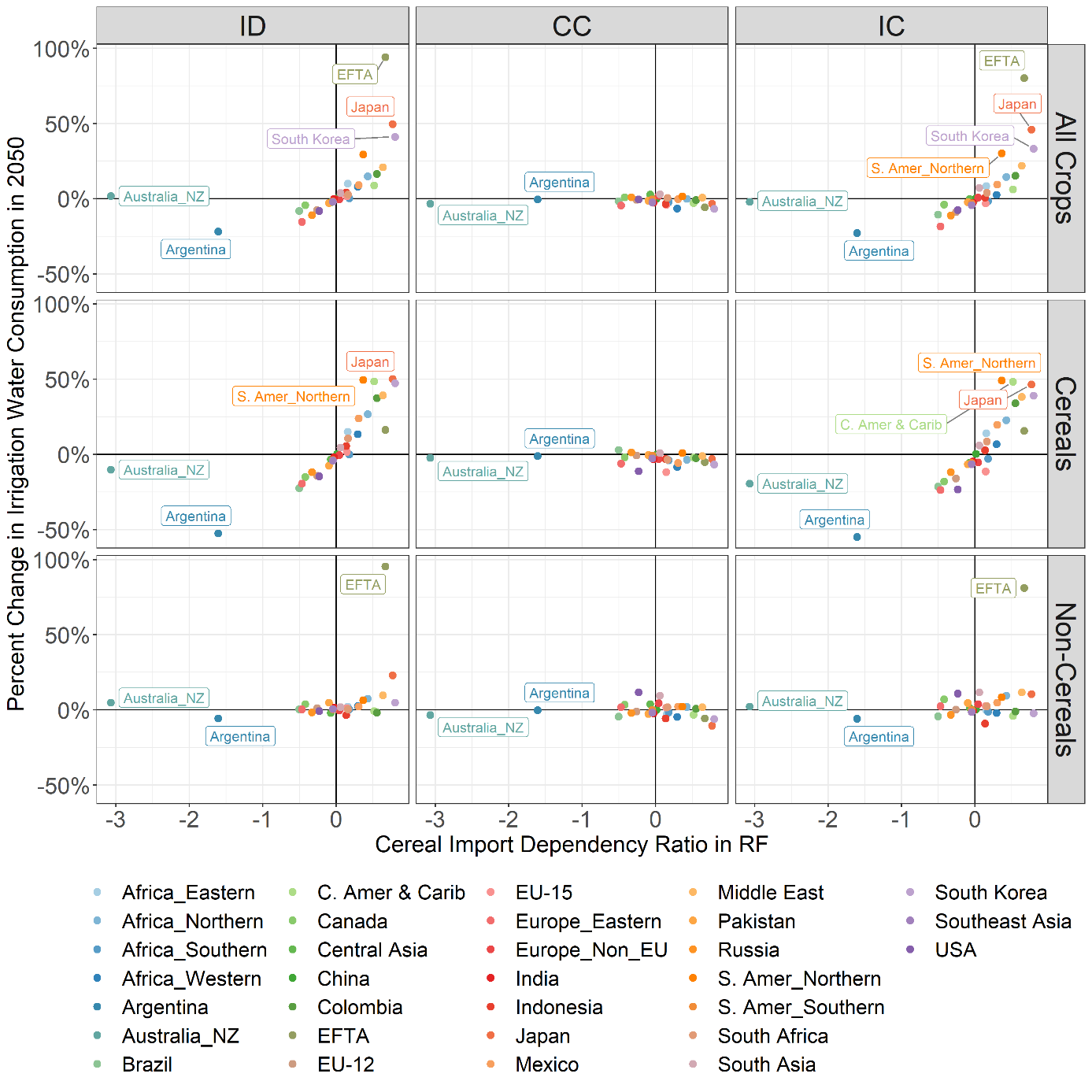


**S6 Fig. Percent change of irrigation water consumption** (km^3^) in 2050 under ID, CC, and IC, compared to the values in 2050 under RF, at the regional level. The x-axis shows the associated cereal import dependency ratio in each region in 2050 under RF. Note that “EFTA” is short for “European Free Trade Association”; “C. Amer & Carib” is short for “Central America and Caribbean”; “S. Amer" is short for “South America”.


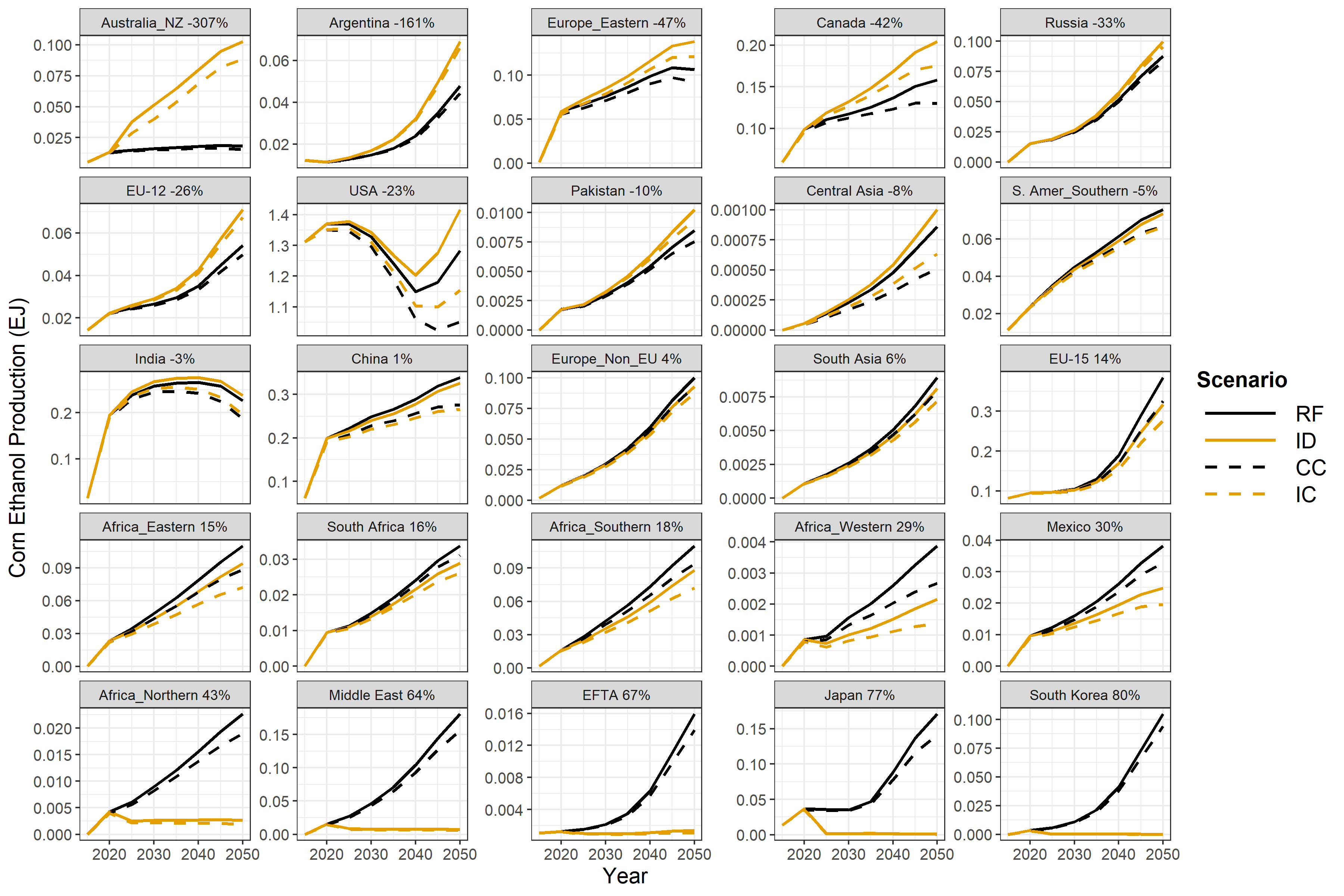


**S7 Fig. Corn ethanol production** (EJ) over 2015-2050 under four model scenarios, across all GCAM regions where corn ethanol is available. The percentage after the region name is the cereal import dependency ratio in 2050 RF, by which the regions are ranked from the smallest to the largest. Please note that because the corn ethanol productions are very different in scale across regions, we use different y-scales for different regions. In addition, in some regions the baseline production in RF is very small which would lead to an extremely large percent change under other scenarios. Therefore, we choose to show the actual corn ethanol production values over time instead of a percent change compared across regions as in other figures (e.g., Figure S6). Note that “EFTA” is short for “European Free Trade Association”; “S. Amer" is short for “South America”.


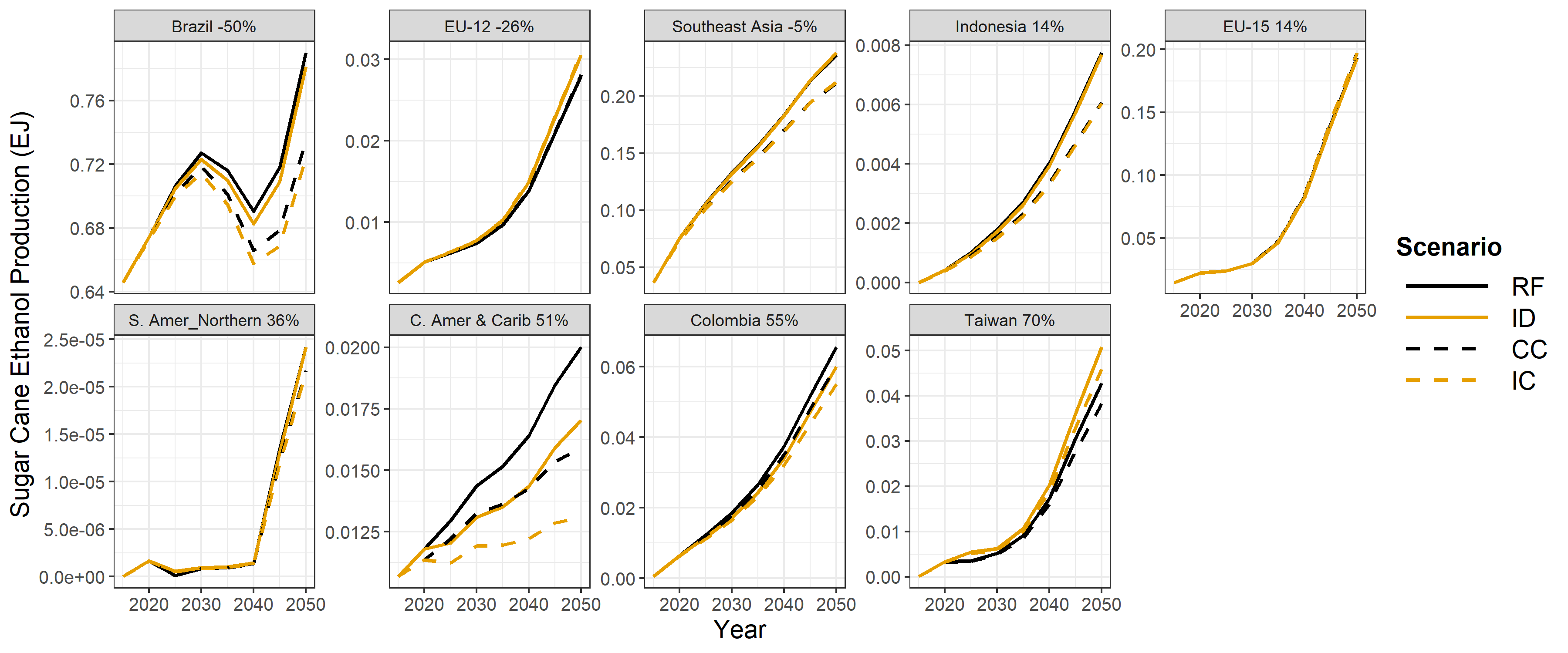


**S8 Fig. Sugarcane ethanol production** (EJ) over 2015-2050 under four model scenarios, across all GCAM regions where sugarcane ethanol is available. The percentage after the region name is the cereal import dependency ratio in 2050 RF, by which the regions are ranked from the smallest to the largest. Please note that because the sugarcane ethanol productions are very different in scale across regions, we use different y-scales for different regions. In addition, in some regions the baseline production in RF is very small which would lead to an extremely large percent change under other scenarios. Therefore, we choose to show the actual sugarcane ethanol production values over time instead of a percent change compared across regions as in other figures (e.g., Figure S6). Note that “C. Amer & Carib” is short for “Central America and Caribbean”; “S. Amer" is short for “South America”.

**S1 Table. GCAM agricultural commodities in staple and non-staple food demand category**

| Food demand category | GCAM agricultural commodities |
| --- | --- |
| Staple | corn, rice, wheat, other grains, roots and tubers |
| Non-staple | sugar crops, fiber crops, soybean, other oil crops, miscellaneous crops, palm fruit, dairy, beef, pork, poultry, fish, sheep and goat |

**S2 Table. Mapping from GCAM region to country**

| GCAM Region | Countries |
| --- | --- |
| Africa_Eastern | Burundi, Comoros, Djibouti, Eritrea, Ethiopia, Kenya, Madagascar, Mauritius, Reunion, Rwanda, Sudan, Somalia, Uganda |
| Africa_Northern | Algeria, Egypt, Western Sahara, Libya, Morocco, Tunisia |
| Africa_Southern | Angola, Botswana, Lesotho, Mozambique, Malawi, Namibia, Swaziland, Tanzania, Zambia, Zimbabwe |
| Africa_Western | Benin, Burkina Faso, Central African Republic, Côte d'Ivoire, Cameroon, Democratic Republic of the Congo, Congo, Cape Verde, Gabon, Ghana, Guinea, Gambia, Guinea-Bissau, Equatorial Guinea, Liberia, Mali, Mauritania, Niger, Nigeria, Senegal, Sierra Leone, Sao Tome and Principe, Chad, Togo |
| Argentina | Argentina |
| Australia_NZ | Australia, New Zealand |
| Brazil | Brazil |
| Canada | Canada |
| Central America and the Caribbean | Aruba, Anguilla, Netherlands Antilles, Antigua & Barbuda, Bahamas, Belize, Bermuda, Barbados, Costa Rica, Cuba, Cayman Islands, Dominica, Dominican Republic, Guadeloupe, Grenada, Guatemala, Honduras, Haiti, Jamaica, Saint Kitts and Nevis, Saint Lucia, Montserrat, Martinique, Nicaragua, Panama, El Salvador, Trinidad and Tobago, Saint Vincent and the Grenadines |
| Central Asia | Armenia, Azerbaijan, Georgia, Kazakhstan, Kyrgyzstan, Mongolia, Tajikistan, Turkmenistan, Uzbekistan |
| China | China |
| Colombia | Colombia |
| EU-12 | Bulgaria, Cyprus, Czech Republic, Estonia, Hungary, Lithuania, Latvia, Malta, Poland, Romania, Slovakia, Slovenia |
| EU-15 | Andorra, Austria, Belgium, Denmark, Finland, France, Germany, Greece, Greenland, Ireland, Italy, Luxembourg, Monaco, Netherlands, Portugal, Sweden, Spain, United Kingdom |
| Europe_Eastern | Belarus, Moldova, Ukraine |
| European Free Trade Association | Iceland, Norway, Switzerland |
| Europe_Non_EU | Albania, Bosnia and Herzegovina, Croatia, Macedonia, Montenegro, Serbia, Turkey |
| India | India |
| Indonesia | Indonesia |
| Japan | Japan |
| Mexico | Mexico |
| Middle East | United Arab Emirates, Bahrain, Iran, Iraq, Israel, Jordan, Kuwait, Lebanon, Oman, Palestine, Qatar, Saudi Arabia, Syria, Yemen |
| Pakistan | Pakistan |
| Russia | Russia |
| South Africa | South Africa |
| South America_Northern | French Guiana, Guyana, Suriname, Venezuela |
| South America_Southern | Bolivia, Chile, Ecuador, Peru, Paraguay, Uruguay |
| South Asia | Afghanistan, Bangladesh, Bhutan, Sri Lanka, Maldives, Nepal |
| Southeast Asia | American Samoa, Brunei Darussalam, Cocos (Keeling) Islands, Cook Islands, Christmas Island, Fiji, Federated States of Micronesia, Guam, Cambodia, Kiribati, Lao People’s Democratic Republic, Marshall Islands, Myanmar, Northern Mariana Islands, Malaysia, Mayotte, New Caledonia, Norfolk Island, Niue, Nauru, Pacific Islands Trust Territory, Pitcairn Islands, Philippines, Palau, Papua New Guinea, Democratic People’s Republic of Korea, French Polynesia, Singapore, Solomon Islands, Seychelles, Thailand, Tokelau, Timor Leste, Tonga, Tuvalu, Viet Nam, Vanuatu, Samoa |
| South Korea | South Korea |
| Taiwan | Taiwan |
| USA | United States |

S3 Table. Major importers and exporters of cereal crops in the reference base year of 2015.

|  | Major Importers | Major Exporters |
| --- | --- | --- |
| **Corn** | Middle East, EU-15, Japan | Brazil, USA, Argentina |
| **Other Grain** | Middle East | Australia_NZ, Argentina |
| **Rice** | Africa_Western, Middle East | Southeast Asia, India |
| **Wheat** | Middle East, Africa_Western, Africa_Northern, Africa_Eastern | USA, Australia_NZ, Canada |

S4 Table. Summary of key variable results in 2050 for Brazil. Note: there is no corn ethanol in Brazil in the model. Instead, sugarcane ethanol is shown in this table.

| **Key Variable** | ***of*** | **RF** | **ID** | **CC** | **IC** |
| --- | --- | --- | --- | --- | --- |
| **Import Dependency Ratio** | *Cereals* | -50% | 0% | -50% | 0% |
| **Production (Mt)** | *Cereals* | 153 | 120 | 145 | 114 |
|  | *All Crops* | 1407 | 1397 | 1371 | 1363 |
| **Consumption (Mt)** | *Cereals* | 101 | 120 | 96 | 114 |
|  | *All Crops* | 1138 | 1138 | 1104 | 1102 |
| **Exports (Mt)** | *Cereals* | 61 | 16 | 58 | 17 |
|  | *All Crops* | 295 | 291 | 293 | 294 |
| **Imports (Mt)** | *Cereals* | 9 | 16 | 10 | 17 |
|  | *All Crops* | 26 | 33 | 26 | 33 |
| **Net Trade Revenue (billion 1975$)** | *Cereals* | 2.23 | -0.06 | 2.25 | -0.03 |
|  | *All Crops* | 12.62 | 11.04 | 13.41 | 11.88 |
| **Crop producers’ Revenue (billion 1975$)** | *Cereals* | 7 | 5 | 7 | 5 |
|  | *All Crops* | 43 | 41 | 44 | 42 |
| **Caloric Consumption (Kcal/cap/day)** | *Cereals* | 1122 | 1163 | 1116 | 1161 |
|  | *Staples* | 1257 | 1258 | 1257 | 1257 |
|  | *All Food* | 3621 | 3623 | 3617 | 3619 |
| **Food Expenditures as a share of income** | *All Food* | 0.72% | 0.71% | 0.74% | 0.73% |
| **Irrigation Water Consumption (km^3^)** | *Cereals* | 5.8 | 4.5 | 6.0 | 4.6 |
|  | *Non-Cereals* | 9.8 | 9.8 | 9.3 | 9.4 |
|  | *All Crops* | 15.6 | 14.3 | 15.3 | 13.9 |
| **Ethanol Production (EJ)** | *Sugarcane* | 0.79 | 0.78 | 0.74 | 0.72 |
| **Sugarcane Consumption for Ethanol (Mt)** | | 460 | 455 | 428 | 421 |
| **Share of Sugar Crop Consumption for Ethanol** | | 52.3% | 52.6% | 50.8% | 50.9% |

In Brazil, the cereal import dependency ratio is -50% in 2050 RF; that is, Brazil is a net exporter of cereals in 2050 RF (Table S2). Under the impact of global zero cereal import dependency (ID), in 2050, the production of cereals decreases 21% and the production of non-cereal crops increases 1.8%. Meanwhile, the export of non-cereal crops in Brazil increases 17.5%, as the rest of world demands more non-cereal crops on average when import of cereals is constrained under ID. Among the non-cereal crops, the domestic sugarcane production increases 2% in Brazil in 2050 ID, with the export of sugarcane increasing 26.8% and the domestic consumption of sugarcane decreasing 1.5%. Therefore, although a higher share of domestic sugarcane is consumed for ethanol production, the resultant ethanol production is lower in ID than RF (Table S2).

S5 Table. Summary of key variable results in 2050 for Western Africa.

| **Key Variable** | ***of*** | **RF** | **ID** | **CC** | **IC** |  |
| --- | --- | --- | --- | --- | --- | --- |
| **Import Dependency Ratio** | *Cereals* | 29% | 0% | 32% | 0% |  |
| **Production (Mt)** | *Cereals* | 99 | 111 | 91 | 103 |  |
|  | *All Crops* | 769 | 829 | 774 | 837 |  |
| **Consumption (Mt)** | *Cereals* | 140 | 111 | 133 | 103 |  |
|  | *All Crops* | 836 | 863 | 843 | 874 |  |
| **Exports (Mt)** | *Cereals* | 0 | 10 | 0 | 10 |  |
|  | *All Crops* | 9 | 19 | 8 | 18 |  |
| **Imports (Mt)** | *Cereals* | 41 | 10 | 42 | 10 |  |
|  | *All Crops* | 76 | 53 | 78 | 55 |  |
| **Net Trade Revenue (billion 1975$)** | *Cereals* | -2.63 | -0.04 | -2.67 | 0.01 |  |
|  | *All Crops* | -2.45 | 0.03 | -2.57 | -0.01 |  |
| **Crop producers’ Revenue (billion 1975$)** | *Cereals* | 12 | 14 | 11 | 14 |  |
|  | *All Crops* | 100 | 107 | 102 | 109 |  |
| **Caloric Consumption (Kcal/cap/day)** | *Cereals* | 876 | 735 | 853 | 693 |  |
|  | *Staples* | 1300 | 1300 | 1300 | 1300 |  |
|  | *All Food* | 2535 | 2534 | 2531 | 2530 |  |
| **Food Expenditures as a share of income** | *All Food* | 2.72% | 2.79% | 2.79% | 2.86% |  |
| **Irrigation Water Consumption (km^3^)** | *Cereals* | 7.1 | 8.0 | 6.5 | 7.5 | |
|  | *Non-Cereals* | 6.6 | 6.7 | 6.3 | 6.4 | |
|  | *All Crops* | 13.6 | 14.7 | 12.7 | 14.0 | |
| **Ethanol Production (EJ)** | *Corn* | 0.004 | 0.003 | 0.002 | 0.001 | |
| **Corn Consumption for Ethanol (Mt)** | | 0.43 | 0.24 | 0.30 | 0.24 | |
| **Share of Corn Consumption for Ethanol** | | 1.31% | 0.76% | 1.02% | 0.76% | |
